# Efficacy and safety of *Ginkgo biloba* leaf extract injection for vascular cognitive impairment: a systematic review and meta-analysis

**DOI:** 10.3389/fphar.2026.1720444

**Published:** 2026-04-13

**Authors:** Jingchao Miao, Yaoyuan Liu, Huichuan Feng, Shujie Zan, Changhong Miao, Jiabao Wang, Xinyao Jin, Fengwen Yang, Wentai Pang

**Affiliations:** 1 Evidence-Based Medicine Center, Tianjin University of Traditional Chinese Medicine, Tianjin, China; 2 Haihe Laboratory of Modern Chinese Medicine, Tianjin University of Traditional Chinese Medicine, Tianjin, China; 3 First Teaching Hospital of Tianjin University of Traditional Chinese Medicine, National Clinical Research Center for Chinese Medicine, Tianjin, China; 4 Traditional Chinese Medical College, North China University of Science and Technology, Tangshan, Hebei, China

**Keywords:** Ginkgo biloba leaf extract injection, Shuxuening injection, vascular cognitive impairment, systematic review, meta-analysis

## Abstract

**Background:**

Shuxuening injection (SXNI), a standardized injectable formulation of *Ginkgo biloba* leaf extract, is widely used in China for vascular cognitive impairment (VCI), However, evidence-based evaluation remains limited.

**Methods:**

Randomized controlled trials published before 1 September 2025, were searched in eight databases. Patients had vascular cognitive impairment (VCI), with the control group receiving conventional treatment and the treatment group receiving additional SXNI therapy. The primary outcome was the Mini-Mental State Examination (MMSE), while secondary outcomes included the Hasegawa Dementia Scale (HDS), Barthel Index (BI), overall response rate, National Institutes of Health Stroke Scale (NIHSS), and adverse event incidence. Risk of bias was assessed using the revised Cochrane Risk of Bias tool. Risk ratios (RR) were used for binary variables; mean differences (MD) with 95% confidence intervals (CI) were used for continuous variables.

**Results:**

Twenty-two trials (n = 2,375) were included. All trials were conducted in China between 2005 and 2025. Compared with the control group, SXNI was associated with higher MMSE (MD 3.61, 95% CI 3.06–4.17), HDS (MD 1.30, 95% CI 0.21–2.39), and BI (MD 9.06, 95% CI 4.66–13.45), a higher overall response rate (RR 1.27, 95% CI 1.21–1.33), and lower NIHSS (MD −6.17, 95% CI −7.90 to −4.45). Seven studies reported adverse events; and there was no significant difference in the incidence of adverse events (RR 0.72, 95% CI 0.36–1.44).

**Conclusion:**

The addition of SXNI to conventional treatment may provide additional benefits in cognition, neurological function, and activities of daily living with no significant increase in reported adverse events compared with conventional treatment. However, the certainty of evidence is limited by methodological weaknesses and uncertainty regarding the comparability of preparations from different manufacturers. Therefore, high-quality studies are needed to confirm these findings.

**Systematic Review Registration:**

https://www.crd.york.ac.uk/PROSPERO/display_record.php?RecordID=557472, identifier CRD42024557472.

## 1 Introduction

Vascular cognitive impairment (VCI) refers to cognitive dysfunction mainly caused by cerebrovascular disease and its risk factors, ranging from subtle cognitive decline to dementia (van der Flier et al., 2018; Iadecola et al., 2019). It can be caused by cerebral small vessel disease, large artery infarctions, and chronic cerebral hypoperfusion (Iadecola, 2013). An analysis for the Global Burden of Disease Study 2019 revealed that dementia affected approximately 57.4 million people globally in 2019, with projections indicating a dramatic increase to 152.8 million cases by 2050 (Collaborators, 2022). Among these, cognitive impairment of vascular etiology is the second most common cause of clinically diagnosed dementia globally, accounting for estimated 20%–40% of all dementia cases (Medina-Rioja et al., 2024). With the population aging and rising prevalence of cerebrovascular disease, the global disease burden of VCI is expected to rise substantially, presenting a significant challenge to public health (Venkat et al., 2015; Mok et al., 2024).

Current treatments for VCI focus primarily on controlling vascular risk factors such as hypertension, diabetes, and dyslipidemia (Skrobot et al., 2018; Ip et al., 2024; Mok et al., 2024; Swartz et al., 2025). Given the pathological and neurochemical overlap between VCI and Alzheimer’s disease (AD), clinicians frequently repurpose the two classes of drugs approved for AD—cholinesterase inhibitors and non-competitive N-methyl-D-aspartate (NMDA) receptor antagonists—as first-line therapy for VCI. These agents have been shown to improve cognitive function and neuropsychiatric symptoms, but they may also increase the incidence of adverse reactions (McShane et al., 2019; Smith et al., 2020; Battle et al., 2021). To date, no drug has been specifically approved for VCI (Gorelick et al., 2011; O'Brien and Thomas, 2015), underscoring the urgent need to identify effective interventions to enhance therapeutic outcomes.

*Ginkgo biloba* leaf extracts have been widely used in clinical settings, particularly in the treatment of cardiovascular and cerebrovascular diseases (Liu et al., 2024). Widely used in current clinical practice in China, *Ginkgo biloba* leaf preparations are recommended as adjunctive therapy for VCI in the Chinese Guidelines for Diagnosis and Treatment of Vascular Cognitive Impairment (Chinese Stroke Association Vascular Cognitive Impairment Subcommittee, 2024). Shuxuening injection (SXNI) is a prescribed injection of *G. biloba* leaf extract, approved by the National Medical Products Administration of China (Supplementary Table S1 for detailed information of SXNI) (Commission, 2020). It is produced by several pharmaceutical manufacturers and is standardized based on labeled contents of total flavonol glycosides and ginkgolides (Supplementary Table S7 for information on SXNI from the included studies). While GBE is typically administered in clinical trials as oral extracts, such as EGb 761, SXNI is formulated for intravenous administration. Studies indicate that *G. biloba* leaf extracts may exert therapeutic effects in VCI via antioxidant, anti-inflammatory, and neuroprotective actions, while simultaneously enhancing cerebral blood flow and modulating neurotransmitter activity (Nathan, 2000; Saleem et al., 2008; Rocher et al., 2011; Zhang and Xue, 2012; Diamond and Bailey, 2013; Kim et al., 2016).

Several randomized controlled trials (RCTs) indicate that adding SXNI to conventional treatment may improve cognitive function and neuropsychiatric symptoms in patients with VCI and is well tolerated (Zheng et al., 2024; Wang and Li, 2025), indicating it warrants further research as a potential therapeutic agent for VCI. However, there has been no systematic quality assessment or evidence synthesis of published RCTs. Therefore, this study aims to synthesize all available RCTs to conduct a comprehensive systematic review and meta-analysis, thereby supporting clinical and policy decision-making regarding SXNI for VCI, while identifying gaps in current research to provide references for the design of future clinical trials (Zhang et al., 2025).

## 2 Methods

This review was registered in the International Prospective Register of Systematic Reviews (PROSPERO, ID: CRD42024557472) and available from https://www.crd.york.ac.uk/PROSPERO/display_record.php?RecordID=557472. It is reported according to the Preferred Reporting Items for Systematic Reviews and Meta-Analyses 2020 (PRISMA 2020) (Page et al., 2021).

### 2.1 Inclusion criteria

1. Types of studies:RCTs were included.
2. Participants:Patients diagnosed with VCI according to the DSM-V (http://www.dsm5.org) or NINDS-AIREN (National Institute of Neurological Disorders and Stroke-International Association for Research and Education in Neurosciences) (Erkinjuntti, 1994). Detailed information of diagnostic criteria for VCI is provided in Supplementary Table S2. Patients with VCI of any clinical subtype were included in the study, with no restrictions on age, gender, race, or region.
3. Intervention and control:The control group received conventional treatment of VCI, including antiplatelet aggregation, regulation of blood lipids, blood pressure and blood glucose, and other medications promoting microcirculation and improving cognition recommended by guidelines (Chinese Stroke Association Vascular Cognitive Impairment Subcommittee, 2024), included piracetam, butylphthalide, oxiracetam, nimodipine, citicoline, and vinpocetine. The treatment group received SXNI in addition to conventional treatment. No restrictions were imposed on either the dosage or treatment duration for either the treatment or the control group.
4. Outcomes:Primary outcome: Mini-Mental State Examination (MMSE). Secondary outcomes: Hasegawa Dementia Scale (HDS), Barthel Index (BI), National Institutes of Health Stroke Scale (NIHSS), overall response rate and incidence of adverse events. Overall response rate was defined based on the improvement of MMSE scores: After treatment, an increase in the MMSE score relative to baseline of ≥12% was considered effective, whereas an increase of <12% was considered ineffective (Tong, 2019; Wu et al., 2021).

### 2.2 Exclusion criteria

1. Patients with traumatic brain injury, epilepsy, Alzheimer’s disease, cancer, organ failure, or any other severe diseases. 2) Studies treated with other traditional Chinese therapy such as herbal medicine, tuina or acupuncture. 3) Studies with incomplete or inadequate data. 4) Duplicate publications were retained only once.

### 2.3 Search strategy

Eight databases were searched from their inception to 1 September 2025 including four English databases (Web of Science, Pubmed, Embase, and Cochrane Library) and four Chinese databases (China National Knowledge Infrastructure Database, VIP Database for Chinese Technical Periodicals, Wanfang Database, and Chinese Biomedical Literature Database). No language restrictions were applied during the literature search. In addition, grey literature sources and clinical trial registries were screened. Search queries are provided in Supplementary Table S3. Taking the search strategy used in PubMed as an example:

#1 (“Cognitive dysfunction” [Mesh Terms] OR “Cognitive Impairments” [Title/Abstract] OR “Vascular Cognitive Impairments” [Title/Abstract] OR “Mild Cognitive Impairment” [Title/Abstract] OR “Cognitive Decline” [Title/Abstract] OR “Vascular cognitive disorders” OR “Vascular neurocognitive disorder” OR “Vascular mild cognitive impairment” OR “Post stroke cognitive impairment”) OR (“Dementia, vascular” [Mesh Terms] OR “Vascular Dementia” [Title/Abstract] OR “Binswanger Disease” [Title/Abstract] OR “Post stroke dementia” [Title/Abstract]) OR (“Dementia, Multi-Infarct” [Mesh Terms] OR “Multi-Infarct Dementias” [Title/Abstract] OR “Dementia Multi-Infarct” [Title/Abstract] OR “Dementia Multi Infarct” [Title/Abstract]).

#2″*Ginkgo*” [Title/Abstract] OR “*G. biloba*” [Title/Abstract] OR “ginkgolide” [Title/Abstract] OR “Yinxing” [Title/Abstract] OR “Yinxingye” [Title/Abstract] OR “Shuxuening” [Title/Abstract] OR “Shuxuening Injection” [Title/Abstract].

#3“randomized controlled trial” [Publication type] OR randomized clinical trial [Publication type] OR randomized trial [Publication type] OR clinical trial [Publication type] OR “randomized controlled trial” [Title/Abstract] OR randomized clinical trial [Title/Abstract] OR randomized trial [Title/Abstract] OR clinical trial [Title/Abstract].

#4 #1 AND #2 AND #3.

### 2.4 Literature screening and data extraction

Literature was managed using EndNote X9. Two researchers independently screened and selected the studies according to the inclusion and exclusion criteria. Disagreements were resolved through discussion with a third researcher. Data were extracted from the eligible studies with a standardized form, collecting the following information: title, first author, publication year, patient characteristics, type of cognitive impairment, drugs used in the intervention and control groups, dosage, treatment duration, and outcomes.

### 2.5 Risk of bias and certainty of evidence assessment

Using the revised Cochrane Risk of Bias tool (ROB2.0) (Sterne et al., 2019), two reviewers independently assessed the risk of bias of the included studies. The assessment covered selection bias, performance bias, detection bias, attrition bias, reporting bias and overall bias. The risk of bias in each domain was classified into three levels: low risk, some concerns and high risk. Grading of Recommendation, Assessment, Development, and Evaluation (GRADE) was used to assess the certainty of evidence for each outcome (Guyatt et al., 2008). Detailed assessment rules are provided in Supplementary Table S4.

### 2.6 Data synthesis and analysis

Statistical analysis of the included studies were conducted using RevMan 5.4 Software. Relative risk (RR) was used to analyze binary variables, while mean difference (MD) was applied for continuous variables, based on a 95% confidence interval (CI). When heterogeneity was low (I^2^<50%), the results would be pooled using a fixed-effect model. When significant heterogeneity was present (I^2^ ≥ 50%), sensitivity analyses or subgroup analyses would be performed to explore potential sources of heterogeneity. If sources of heterogeneity remained unidentified, data would be pooled using a random-effects model. For publication bias assessment, funnel plots were generated when the number of included studies exceeded 10.

## 3 Results

### 3.1 Results of study selection

**FIGURE 1:**
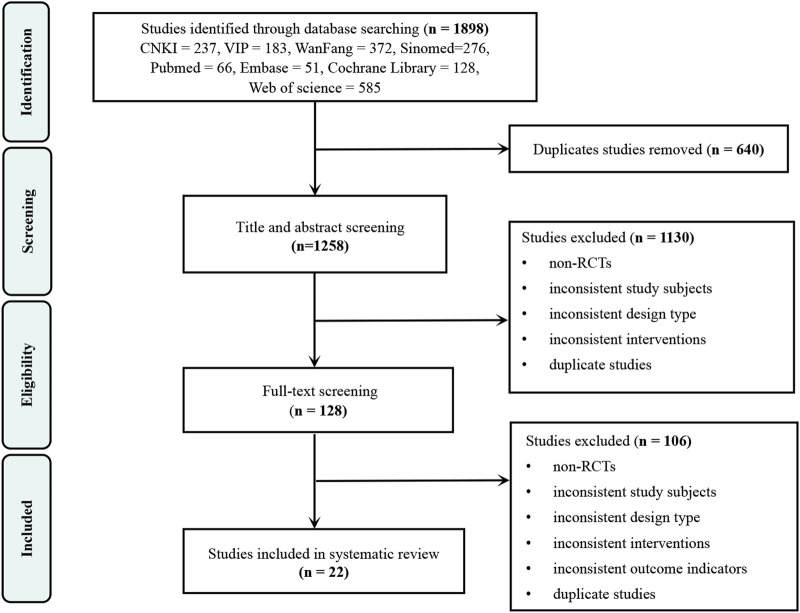
Study selection process. Studies were excluded at the full-text screening stage due to non-RCTs, inconsistent study subjects, inconsistent design type, inconsistent interventions, inconsistent outcome indicators, or duplicate studies. Abbreviations: CNKI, China National Knowledge Infrastructure Database; VIP, VIP Database for Chinese Technical Periodicals; RCTs, randomized controlled trials.

A total of 1,898 studies were searched. After removing 640 duplicates, 1,258 studies remained. Screening of titles and abstracts excluded a further 1,130 studies, leaving 128 for full-text assessment. Of these, 106 were excluded, resulting in 22 studies being included. The study selection process is illustrated in Figure 1.

### 3.2 Characteristics of the included studies

A total of 22 RCTs involving 2,375 patients were included, with 1,193 patients in the treatment group and 1,182 in the control group. All trials were conducted in China from 2005 to 2025. The mean age of the participants ranged from 57.47 to 83.60 years. The control groups received conventional treatment, which included antiplatelet aggregation, regulation of blood lipids, blood pressure and blood glucose, and other medications promoting microcirculation and improving cognition, included piracetam, butylphthalide, oxiracetam, nimodipine, citicoline, and vinpocetine. The treatment group was treated with SXNI administered intravenously in addition to the conventional treatment. The daily dosage of SXNI ranged from 10 mL to 50 mL, and the duration of treatment ranged from 2 weeks to 3 months. There were no significant differences found between the baselines described in any of the studies. The characteristics of the included studies were indicated in Table 1.

**TABLE 1:** Characteristics of the included studies.

Included studies	Types of cognitive impairment	Sample size		Age (mean ± standard deviation)		Gender (M/F)		Intervention		Treatment duration	Outcomes
		T	C	T	C	T	C	T	C		
Hu (2005)	MID	68	66	67.9 ± 10.07	68.1 ± 10.28	48/20	47/19	SXNI 20 mL QD + Piracetam 0.8 g TID	Piracetam 0.8 g TID	14d	④⑥
Zuo (2006)	VD	50	50	NK	NK	38/12	12/14	SXNI 50 mL QD + Citicoline 0.5 g, Venoruton 0.5 g QD	Citicoline 0.5 g, Venoruton 0.5 g QD	45d	①③
Zhou and Zhang (2007)	VD	28	26	67.5	66.7	16/12	15/11	SXNI 20 mL QD + Citicoline1 g QD	Citicoline1 g QD	14d	④
Hu (2009)	VD	59	59	76.5 ± 5.9	77.6 ± 6.0	31/28	30/29	SXNI 20 mL QD + Nimodipine 40 mg TID	Nimodipine 40 mg TID	30d	①④⑥
Hu et al. (2012)	VD	46	46	72.46 ± 9.32	72.46 ± 9.32	28/18	28/18	SXNI 15 mL QD + Piracetam 100 mL QD BID	Piracetam 100 mL QD BID	21d	①
Yang and Liang (2014)	VD	31	31	61.81 ± 7.37	61.81 ± 7.37	NK	NK	SXNI 20 mL QD + Citicoline 1 g QD	Citicoline 1 g QD	28d	①④⑥
Su and Shang (2014)	PSCI	91	91	57.47	57.47	51/40	51/40	SXNI 20 mL QD + CT	CT	14d	①④
Yang (2015)	VD	29	29	62.3 ± 1.7	62.3 ± 1.7	16/13	16/13	SXNI 20 mL QD + Cattle Encephalon Glycoside and Ignotin Injection 10 mL QD	Cattle Encephalon Glycoside and Ignotin Injection 10 mL QD	14d	①④⑥
Chen (2016)	VD	38	38	72.8 ± 8.2	73.1 ± 8.3	20/18	21/17	SXNI 15 mL QD + Piracetam 100 mL QD	Piracetam 100 mL QD	28d	①④
Li et al. (2017)	VD	40	40	70 ± 8.5	70 ± 8.5	20/20	20/20	SXNI 20 mL QD + Butylphthalide 0.2 g TID	Butylphthalide 0.2 g TID	56d	①②④
Pang et al. (2017)	VD	52	52	68.9 ± 4.3	68.1 ± 3.8	28/24	29/23	SXNI 15 mL QD + Piracetam 20 mL BID	Piracetam 20 mL BID	21d	①④
Hu (2018)	VD	50	50	77.1 ± 4.7	69.4 ± 5.7	29/21	27/23	SXNI 20 mL QD + Nimodipine 30 mg TID	Nimodipine 30 mg TID	30d	①③④
Peng (2018)	VCI	142	142	74.81 ± 4.97	74.25 ± 5.02	78/64	80/62	SXNI 20 mL QD + Vinpocetine20 mg QD	Vinpocetine20 mg QD	14d	①②
Tong (2019)	VD	43	43	65.76 ± 6. 98	65. 69 ± 7. 06	24/19	23/20	SXNI 20 mL QD + Butylphthalide 0.2 g TID	Butylphthalide 0.2 g TID	30d	①③④⑥
Weng (2019)	VD	95	90	69.5 ± 6.8	70.2 ± 7.4	49/46	46/44	SXNI 20 mL QD + Oxiracetam Injection 4 g QD	Oxiracetam Injection 4 g QD	28d	①④⑤
Yang (2020)	VD	80	80	74.23 ± 4.61	74.12 ± 3.41	49/31	46/34	SXNI 20 mL QD + Piracetam 0.8 g TID	Piracetam 0.8 g TID	60d	①③④
Wang (2021)	VD	49	51	69.21 ± 3.21	68.34 ± 3.56	25/24	29/22	SXNI 20 mL QD + Butylphthalide 0.2 g TID	Butylphthalide 0.2 g TID	60d	①②③④⑥
Wu et al. (2021)	PSD	30	30	63.86 ± 5.12	64.38 ± 4.95	18/12	17/13	SXNI 20 mL QD + Donepezil 5 mg QD	Donepezil 5 mg QD	30d	①④
Luan (2021)	PSCI	40	40	69.40 ± 6.70	69.40 ± 6.70	20/20	21/19	SXNI 20 mL QD + Oxiracetam Injection 4 g QD	Oxiracetam Injection 4 g QD	21d	①④
Jiang (2024)	VD	52	48	72.5 ± 4.1	71.2 ± 3.9	NK	NK	SXNI 20 mL QD + Butylphthalide 0.2 g	Butylphthalide 0.2 g TID	56d	①④
Zheng et al. (2024)	VD	40	40	73.58 ± 8.29	70.03 ± 9.35	21/19	20/20	SXNI 20 mL QD + Idebenone 30 mg TID	Idebenone 30 mg TID	7d	①③④
Wang and Li (2025)	VD	40	40	72.34 ± 6.14	72.45 ± 6.22	23/17	24/16	SXNI 10 mL QD + Butylphthalide 25 mg BID + Memantine 5 mg QD	Butylphthalide 25 mg BID + Memantine 5 mg QD	30d	①④⑤⑥

Abbreviations: T, treatment group; C, control group; d, days; SXNI, shuxuening injection; CT, conventional treatment; QD, once a day; BID, twice a day; TID, three times a day; NK, not known; ①Mini-Mental State Examination; ②Hasegawa Dementia Scale; ③Barthel Index; ④overall response rate; ⑤National Institutes of Health Stroke Scale; ⑥incidence of adverse events.

### 3.3 Risk of bias

**FIGURE 2:**
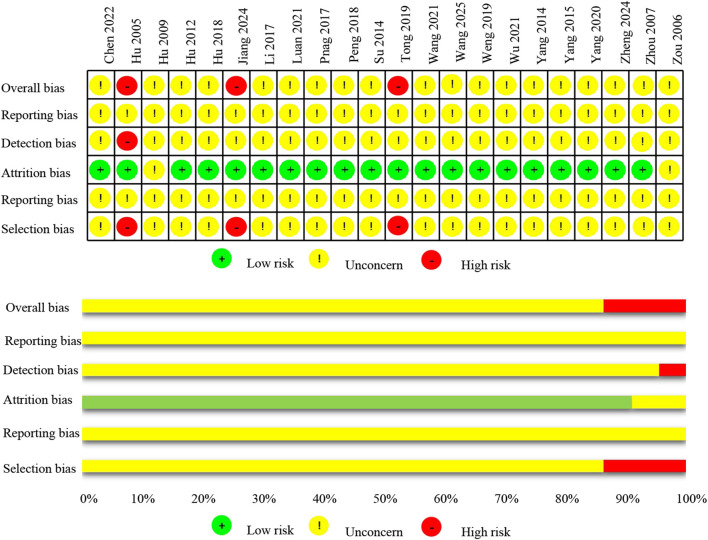
Risk of bias of included studies.

1. All studies claimed to have conducted random allocation. Two of the studies used inappropriate randomization methods with unclear allocation concealment rated as high risk. One study did not conduct allocation concealment was rated as high risk. The remaining nineteen studies with balanced baseline characteristics were rated as having some concerns due to unclear allocation concealment. (2) None of the studies reported the use of blinding, so all studies were rated as some concerns in terms of performance bias. (3) Twenty studies had complete data with no missing information, therefore, the attrition bias was rated as low risk. Two study did not report dropouts were rated as some concerns. (4) None of the studies reported the use of blinding, and one of them potentially employed inappropriate methods for measuring outcomes, thus being rated as high risk for detection bias. The remaining studies were rated as some concerns. (5) Among the included studies, all reported on the intended outcomes. However, since none of the study reported a pre-published research protocol, the reporting bias was rated as some concerns. Finally, the overall bias for nineteen studies were rated as some concerns, and three studies were rated as high risk. The assessment of the risk of bias of the included studies is shown in Figure 2.

### 3.4 Outcomes

#### 3.4.1 MMSE

**FIGURE 3:**
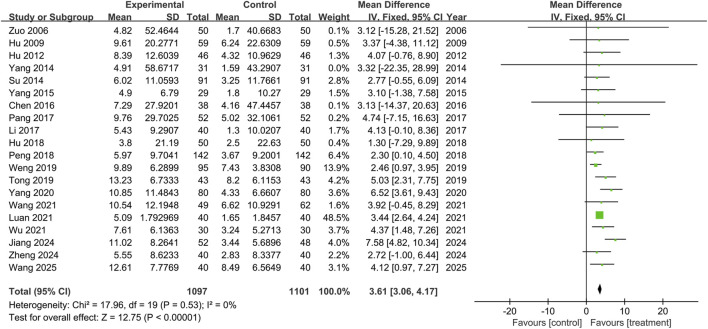
The forest plot of Mini-Mental State Examination.

**FIGURE 4:**
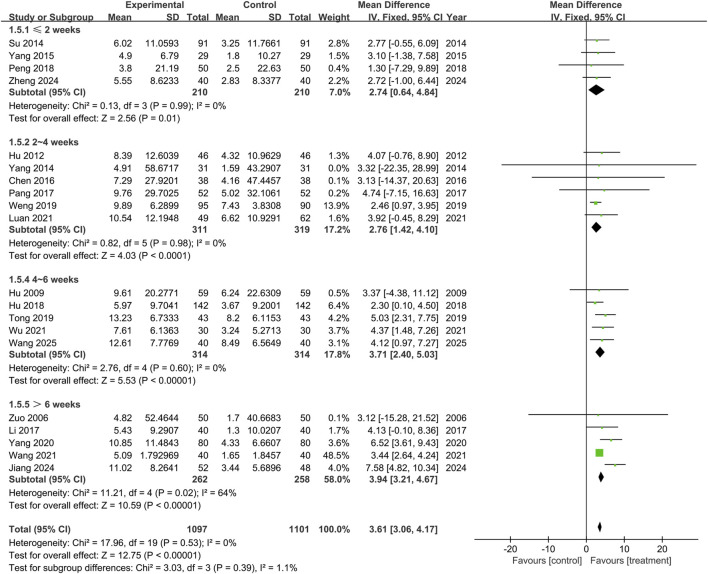
Subgroup analysis of Mini-Mental State Examination based on treatment duration.

This study included twenty trials assessing the MMSE (n = 2,198), and no significant heterogeneity was observed between the results of the studies (P = 0.53, I^2^ = 0%). A fixed-effects model was used for the meta-analysis, and the results showed that the treatment group outperformed the control group in improving MMSE scores, with statistical significance (MD = 3.61, 95% CI: 3.06 to 4.17, P < 0.00001), as shown in Figure 3. Subgroup analyses were conducted according to treatment duration and background medications. Subgroup analysis based on treatment duration showed that the treatment group outperformed the control group in ≤2 weeks subgroup (MD = 2.74, 95% CI: 0.64 to 4.84, P = 0.01), 2∼4 weeks subgroup (MD = 2.76, 95% CI: 1.42 to 4.10, P < 0.0001), 4∼6 weeks subgroup (MD = 3.71, 95% CI: 2.40 to 5.03, P < 0.00001), and >6 weeks subgroup (MD = 3.94, 95% CI: 3.21 to 4.67, P < 0.00001), with the differences being statistically significant. There was no significant difference among the subgroups (P = 0.39), as shown in Figure 4.

**FIGURE 5:**
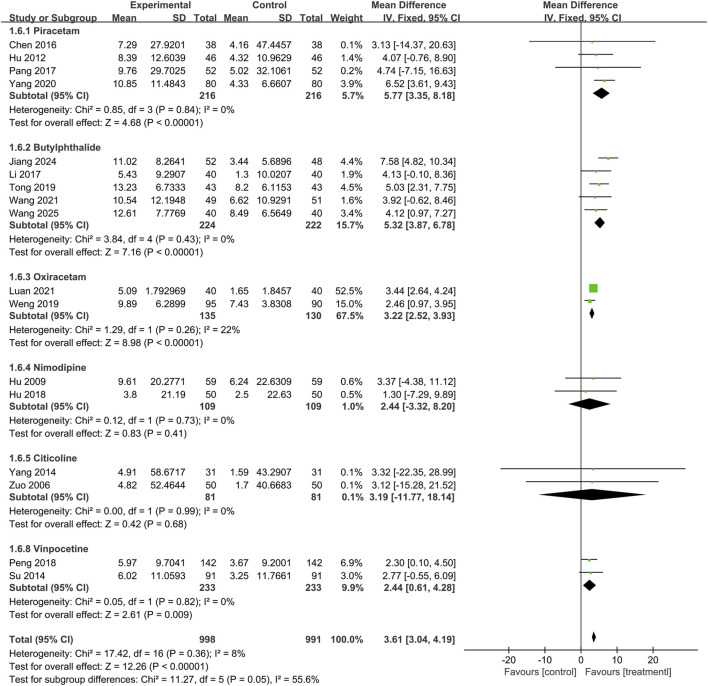
Subgroup analysis of Mini-Mental State Examination based on background medications.

Subgroup analyses were performed according to the background medications, and the results are shown in Figure 5. When the background medication was piracetam (MD = 5.77, 95% CI: 3.35 to 8.18; I^2^ = 0%), butylphthalide (MD = 5.32, 95% CI: 3.87 to 6.78; I^2^ = 0%), oxiracetam (MD = 3.16, 95% CI: 2.29 to 4.03; I^2^ = 22%), or vinpocetine (MD = 2.44, 95% CI: 0.61 to 4.28; I^2^ = 0%), the treatment group demonstrated superior efficacy compared with the control group, and the difference was statistically significant. When the background medication was nimodipine (MD = 2.44, 95% CI: −3.32 to 8.20; I^2^ = 0%) or citicoline (MD = 3.19, 95% CI: −11.77 to 18.14; I^2^ = 0%), the difference in efficacy between the treatment and control groups was not statistically significant. The difference between subgroups was statistically significant (P = 0.05).

#### 3.4.2 HDS

**FIGURE 6:**
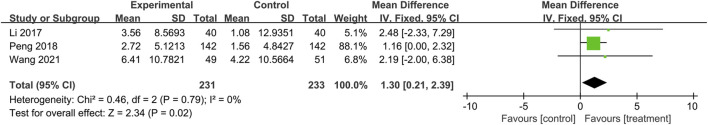
The forest plot of Hasegawa Dementia Scale.

This study included three trials assessing the HDS (n = 464), and no significant heterogeneity was observed between the results of the studies (P = 0.79, I^2^ = 0). A fixed-effects model was used for the meta-analysis, and the results showed that the treatment group outperformed the control group in improving HDS scores, with statistical significance (MD = 1.30, 95% CI: 0.21 to 2.39, p = 0.02), as shown in Figure 6.

#### 3.4.3 Barthel Index

**FIGURE 7:**
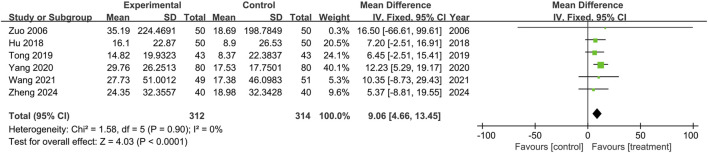
The forest plot of Barthel Index.

This study included six trials assessing the BI (n = 626), and no significant heterogeneity was observed between the results of the studies (P = 0.90, I^2^ = 0). A fixed-effects model was used for the meta-analysis, and the results showed that the treatment group outperformed the control group in improving BI scores, with statistical significance (MD = 9.06, 95% CI: 4.66 to 13.45, p < 0.0001), as shown in Figure 7.

#### 3.4.4 Overall response rate

**FIGURE 8:**
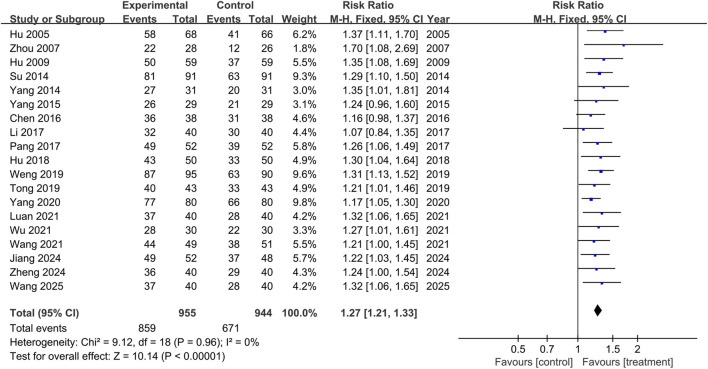
The forest plot of overall response rate.

This study included nineteen trials assessing the overall response rate (n = 1,899), and no significant heterogeneity was observed between the results of the studies (P = 0.96, I^2^ = 0). A fixed-effects model was used for the meta-analysis, and the results showed that the treatment group outperformed the control group in improving overall response rate, with statistical significance (RR = 1.27, 95% CI: 1.21 to 1.33, p < 0.00001), as shown in Figure 8.

#### 3.4.5 NIHSS

**FIGURE 9:**
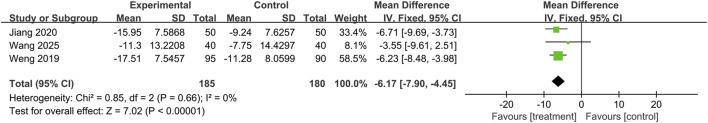
The forest plot of National Institutes of Health Stroke Scale.

This study included three trials assessing the NIHSS (n = 365), and no significant heterogeneity was observed between the results of the studies (P = 0.66, I^2^ = 0). A fixed-effects model was used for the meta-analysis, and the results showed that the treatment group outperformed the control group in reducing NIHSS scores, with statistical significance (MD = −6.17; 95% CI: -7.90 to −4.45; p < 0.00001), as shown in Figure 9.

#### 3.4.6 Adverse events

**FIGURE 10:**
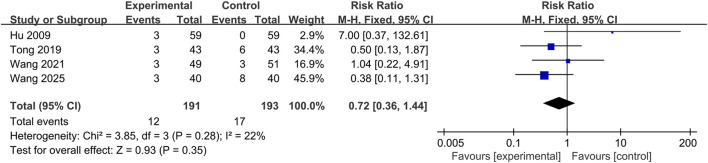
The forest plot of incidence of adverse events.

Seven studies reported the incidence of adverse events, of which three studies reported no adverse events. Three studies reported nausea and vomiting, abdominal discomfort, headache, dizziness, hallucination, and elevated transaminases in both groups. One study reported facial redness, constipation, drowsiness, and diarrhea in the treatment group. None of the studies reported life-threatening adverse events. The occurrence of adverse events is detailed in Table 2. No significant heterogeneity was observed between the results of the studies (P = 0.28, I^2^ = 22%). A fixed-effects model was used for the meta-analysis, and the results showed that there was no significant difference in the incidence of adverse events between the two groups, (RR = 0.72; 95% CI: 0.36 to 1.44; p = 0.35), as shown in Figure 10.

**TABLE 2:** The occurrence of adverse events.

Treatment	Sample size	Nausea/Vomiting	Headache	Abdominal discomfort	Elevated transaminase	Facial redness	Constipation	Drowsiness	Diarrhea	Total adverse events
SXNI + CT	241	4	2	2	1	1	1	1	2	14
CT	243	4	1	3	1	0	0	2	2	13

Abbreviations: SXNI, shuxuening injection; CT, conventional treatment.

#### 3.4.7 Sensitivity analyses

Sensitivity analyses were performed by excluding studies assessed as overall high risk of bias. After exclusion, the pooled efficacy estimates remained largely consistent with the primary analyses. The improvement in MMSE scores remained statistically significant (MD 3.37, 95% CI 2.79–3.95). Similarly, significant benefits were observed for the BI (MD 9.88, 95% CI 4.83–14.93) and overall response rate (RR 1.26, 95% CI 1.20–1.33). For safety outcomes, the pooled estimate for adverse events remained non-significant after exclusion of high-risk studies (RR 0.84, 95% CI 0.37–1.91), although the confidence interval was wide, reflecting limited precision due to the small number of trials reporting adverse events. Overall, these findings indicate that the primary efficacy results were robust to the exclusion of studies at high risk of bias. Detailed results of the sensitivity analyses are provided in Supplementary Material 6.

### 3.5 Publication bias

**FIGURE 11:**
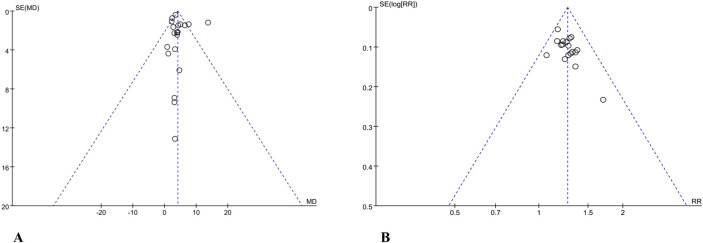
**(A)** Funnel plots of Mini-Mental State Examination. **(B)** Funnel plots of overall response rate.

Publication bias was assessed for MMSE and overall response rate, each involving more than ten studies. Funnel plots revealed a relatively symmetrical distribution of data points, suggesting there may not be substantial publication bias for these outcomes, as illustrated in Figures 11A,B.

### 3.6 Certainty of evidence assessment

The certainty of evidence assessment is presented in Table 3. Overall, the results indicate moderate certainty for BI and overall response rate, low certainty for MMSE and the incidence of adverse events, and very low certainty for HDS and NIHSS.

**TABLE 3:** Certainty of evidence.

Outcomes	Certainty assessment							Effect (95% CI)		Certainty
	No. of studies	Study design	Inconsistency	Indirectness	Imprecision	Publication bias	No. of patients	Relative (RR)	Absolute (MD)	
MMSE	20	Serious^a^	Serious^b^	Not serious	Not serious	Not serious	1288	0.63 [0.52, 0.75]	N/A	⊕⊕○○ Low
HDS	3	Serious^a^	Not serious	Not serious	Serious^c^	N/A	87	N/A	5.28 [-2.12, 12.68]	⊕○○○ Very low
BI	6	Serious^a^	Not serious	Not serious	Not serious	N/A	844	0.60 [0.36, 1.01]	N/A	⊕⊕⊕○ Moderate
Clinical efficacy	18	Serious^a^	Not serious	Not serious	Not serious	Not serious	1411	N/A	−2.22 [-2.89, −1.55]	⊕⊕⊕○ Moderate
NIHSS	2	Serious^a^	Not serious	Not serious	Serious^c^	N/A	78	N/A	10.40 [4.53, 16.27]	⊕○○○ Very low
Incidence of adverse events	7	Serious^a^	Not serious	Not serious	Not serious	N/A	1372	0.65 [0.45, 0.93]	N/A	⊕⊕○○ Low

Abbreviations: CI, confidence intervals; RR, risk ratio; MD, mean difference; MMSE, Mini-Mental State Examination; HDS, hasegawa dementia scale; BI, barthel index; NIHSS, national institutes of health stroke scale.

^a^ Most (two-thirds) of the results came from studies with some concerns or a high risk of overall bias.

^b^ Heterogeneity tests showed I2 exceeding 75%.

^c^ The sample size was significantly smaller than the optimal information size.

+There is significant publication bias.

## 4 Discussion

### 4.1 Summary of key findings

#### 4.1.1 Efficacy

In recent years, there has been growing interest among the public and medical community in the use of *G. biloba* leaf extracts for treating cognitive impairment and dementia (Sachdev et al., 2014; Skrobot et al., 2018). In this systematic review, pooled analyses suggested potential improvements in cognitive function, activities of daily living, and neurological outcomes in patients with vascular cognitive impairment when Ginkgo biloba leaf extract injection was used as adjunctive therapy. No significant difference in adverse event incidence was observed compared to conventional treatment alone. Sensitivity analyses excluding studies at high risk of bias yielded results consistent with the primary analyses. However, interpretation of these findings should be cautious given the substantial methodological limitations of the included trials. Overall, the current evidence indicates a possible benefit, but the certainty of the evidence remains limited.

Clinical trials and meta-analyses worldwide have investigated the efficacy of *G. biloba* leaf extracts, most notably EGb 761, in cognitive disorders (Serge and Sandra, 2014). Meta-analyses have reported modest but statistically significant benefits of oral *G. biloba* leaf extracts on global cognitive function and activities of daily living, particularly in patients with cognitive impairment (Riepe et al., 2025). SXNI is an intravenous botanical drug with a distinct formulation, pharmacokinetic profile, and clinical context compared with oral *G. biloba* leaf preparations used internationally. Moreover, differences in study populations, background therapies, and outcome assessment limit direct comparability. Therefore, evidence from oral *G. biloba* leaf preparations trials should be viewed as complementary.

#### 4.1.2 Safety

Safety reporting in the included trials was limited and heterogeneous. Only seven of the twenty-two studies reported adverse events, and three of these explicitly stated that no adverse events occurred. Reported events were generally mild and included gastrointestinal discomfort, dizziness, headache, and transient elevations of transaminases. No life-threatening events were described. Meta-analysis did not demonstrate a statistically significant difference in overall adverse event incidence between groups. However, the limited number of trials reporting safety outcomes, short follow-up duration (mostly 2 weeks to 3 months), and lack of standardized adverse event monitoring substantially restrict interpretation.

Importantly, potential interactions with anticoagulants or antiplatelet agents, which are clinically relevant in patients with cerebrovascular disease, were not systematically assessed or reported. Therefore, the absence of reported serious adverse events should not be interpreted as evidence of absence of risk.

Real-world studies have provided further evidence of its safety—a post-marketing surveillance study involving 30,122 patients reported an SXNI-related adverse event rate of 0.18% (95% CI: 0.13%–0.23%), with no severe adverse reactions observed, indicating that SXNI-associated adverse effects occur only sporadically (Xinyao et al., 2023). Post-marketing surveillance data have suggested a low incidence of reported adverse reactions; however, such observational data cannot substitute for rigorously designed trials with comprehensive safety monitoring. Overall, the available safety evidence remains limited and preliminary.

### 4.2 Clinical application

Increase in MMSE exceeds the commonly cited minimal clinically important difference (MCID) in patients with cognitive impairment and dementia (Muir et al., 2024). Such an improvement is generally considered indicative of clinically meaningful cognitive change. Besides, the results of the subgroup analysis provide a reference for the clinical application strategy of SXNI. Subgroup analysis stratified by treatment duration (≤2 weeks, 2∼4 weeks, 4∼6 weeks, and >6 weeks) showed a positive trend in cognitive improvement with prolonged treatment. Although the test for subgroup differences did not reach statistical significance (P = 0.39), it still suggests that cognitive improvement may be associated with longer treatment duration. This raises a critical, previously unaddressed question: Is there a critical time window for initiating SXNI therapy post-stroke or post-diagnosis to maximize efficacy?

Subgroup analysis based on background medications revealed that when SXNI was combined with donepezil, butylphthalide, piracetam, or vinpocetine, the improvement in MMSE was statistically significant compared to the control group. However, no significant benefit was observed when SXNI was combined with nimodipine or citicoline. Subgroup analyses suggested that the magnitude of MMSE improvement varied across different concomitant therapies. However, given the heterogeneity of background treatments and the limited sample sizes within subgroups, these findings should be interpreted with caution. For discharged patients who have difficulty receiving intravenous therapy, switching to oral *G. biloba* leaf preparations may also be considered as a supplementary treatment option (Herrschaft et al., 2012; Zhan et al., 2021).

### 4.3 Potential mechanisms

The therapeutic effects of SXNI in VCI attributed to multiple mechanisms of action. At the vascular level, it enhances cerebral blood flow by promoting nitric oxide production and modulating endothelium-dependent relaxation, while simultaneously reducing blood viscosity and improving cerebral microcirculation (Nash and Shah, 2015; Bahdar et al., 2025). Furthermore, it may effectively scavenge reactive oxygen species, upregulates antioxidant enzymes, and suppresses pro-inflammatory cytokines, supporting long-term cognitive preservation (Shi et al., 2010; Singh et al., 2019). It may exert antioxidant effects by protecting mitochondria, as the mitochondrial respiratory chain is both a key source and target of reactive oxygen species (Diamond and Bailey, 2013). Additionally, the neuroprotective mechanisms include preventing neuronal apoptosis, preserving synaptic plasticity, and promoting neurogenesis (Wang and Han, 2015; Zang et al., 2023; Muratori et al., 2025).

An emerging and conceptually appealing way to interpret the potential mechanisms of SXNI is through the lens of network pharmacology. This perspective is informed by existing pharmacological. Unlike single-target synthetic drugs, SXNI contains multiple bioactive metabolites that may simultaneously influence interconnected pathological processes involved in VCI, including vascular endothelial dysfunction, oxidative stress, neuroinflammation, and apoptotic signaling. From a network pharmacology perspective, SXNI could hypothetically contribute to rebalancing these dysregulated biological networks, rather than acting on a single molecular target.

### 4.4 Strengths and limitations

This study has several limitations that should be acknowledged. First, all included studies were conducted in China, which may limit generalizability. Second, the absence of blinding, inadequate reporting of allocation concealment, and lack of publicly available pre-registered protocols in most included trials constitute major methodological limitations, particularly for subjective cognitive outcomes. Additionally, clinical heterogeneity may exist due to the inclusion of different VCI subtypes. Safety conclusions are limited by the small number of trials reporting such outcomes, short follow-up durations, and insufficient reporting of safety monitoring methods. Besides, none of the included studies employed a placebo-controlled design and sample sizes were generally small, which may have increased the risk of overestimating treatment effects. Finally, although available label and regulatory information suggests some degree of standardization across marketed SXNI products, the absence of chemical characterization in included studies means that the comparability of the preparations across studies could not be fully assessed.

Despite these limitations, the study performed subgroup analyses based on treatment duration and background medications, which allowed for a deeper exploration of how different factors influence treatment outcomes. Besides, this study provides comprehensive evidence supporting the use of SXNI as an adjunctive therapy for VCI. The findings offer valuable insights for clinical practice and future research directions.

### 4.5 Future perspectives

Future studies should address the limitations of current evidence by conducting large-scale, multicenter, double-blind RCTs with longer follow-up periods. More precise classification of VCI subtypes and standardization of treatment protocols are needed to enhance the clinical applicability of research findings. Additionally, studies exploring the optimal dosing, treatment duration, and combination strategies for SXNI would provide further guidance for clinical practice.

## 5 Conclusion

Compared with conventional treatment alone, the addition of SXNI may be associated with potential benefits for VCI patients in improving cognitive function, enhancing daily living abilities, and promoting neurological recovery, with no significant increase in reported adverse events compared with conventional treatment. However, the overall certainty of evidence is limited by methodological weaknesses and uncertainty regarding the comparability of preparations from different manufacturers. Therefore, the findings should be interpreted with caution and require confirmation in well-designed, high-quality randomized controlled trials.

## Data Availability

The original contributions presented in the study are included in the article/Supplementary Material, further inquiries can be directed to the corresponding author.
