# Supplementary material for: Efficacy and safety of Ginkgo biloba leaf extract injection for vascular cognitive impairment: a systematic review and meta-analysis
Source: Front Pharmacol. 2026 Apr 13;17:1720444. doi: 10.3389/fphar.2026.1720444 (PMC13111442; doi:10.3389/fphar.2026.1720444)
Supplement: Supplementary file 1 [file Table1.docx]

Supplementary Material

# **Supplementary Table 1 Detailed information on the SXNI**

| Ingredients | Specifications | Usage and dosage | Indication |
| --- | --- | --- | --- |
| total flavonol glycosides; ginkgolides | Every 5 mL SXNI contains 17.5 mg of ginkgo biloba extract (4.2 mg of total flavonoid glycosides and 0.70 mg of ginkgolide) | Intramuscular injection: 2-4 mL per dose, 1-2 times daily.  Intravenous infusion: 20 mL per day, diluted in 250 mL or 500 mL of 5% glucose injection before use | Used for cognitive dysfunction, headache, tinnitus, vertigo, inattention, mood disturbances, cardiovascular disease |

# Supplementary Table 2 Diagnostic criteria for Vascular Cognitive Impairment

①Confirm cognitive symptoms (subjective complaints) and show impairment in ≥1 cognitive domain on neuropsychological testing.

② Establish evidence of cerebrovascular brain injury (e.g., vascular risk factors, stroke/TIA history, focal vascular neurological syndromes, or neuroimaging signs—these need not all be present).

③ vascular injury must be the primary driver of the cognitive syndrome.

④MRI shows changes meeting the VASCOG minimum imaging standards.

# Supplementary Table 3 Search queries for each database

| Database | Search query |  |
| --- | --- | --- |
| CNKI | SU=("舒血宁注射液" + "舒血宁注射剂" + "银杏叶提取物" + "银杏叶注射液" + "银杏叶制剂" + "银杏叶注射剂" + "舒血宁") AND SU=("认知障碍" + "血管性痴呆" + "卒中后痴呆" + "认知功能障碍" + "混合性痴呆" + "动脉硬化性痴呆" + "丘脑性痴呆" + "[关键部位](https://baike.baidu.com/item/%E5%85%B3%E9%94%AE%E9%83%A8%E4%BD%8D/8678986?fromModule=lemma_inlink)梗死性痴呆" + "出血性痴呆" + "多发性梗死痴呆" + "多发性硬化性痴呆" + "多发性梗塞痴呆" + "宾斯旺格病" ) AND SU=("临床"+"疗效"+"随机"+"观察"+"评价"+"安全性"+"有效性") |  |
| VIP | M=("舒血宁注射液" + "舒血宁注射剂" + "银杏叶提取物" + "银杏叶注射液" + "银杏叶制剂" + "银杏叶注射剂" + "舒血宁") AND M=("认知障碍" + "血管性痴呆" + "卒中后痴呆" + "认知功能障碍" + "混合性痴呆" + "动脉硬化性痴呆" + "丘脑性痴呆" + "[关键部位](https://baike.baidu.com/item/%E5%85%B3%E9%94%AE%E9%83%A8%E4%BD%8D/8678986?fromModule=lemma_inlink)梗死性痴呆" + "出血性痴呆" + "多发性梗死痴呆" + "多发性硬化性痴呆" + "多发性梗塞痴呆" + "宾斯旺格病" ) AND M=("临床"+"疗效"+"随机"+"观察"+"评价"+"安全性"+"有效性") |  |
| Wanfang | 主题:("舒血宁注射液" OR "舒血宁注射剂" OR "银杏叶提取物" OR "银杏叶注射液" OR "银杏叶制剂" OR "银杏叶注射剂" OR "舒血宁") AND 主题:("认知障碍" OR "血管性痴呆" OR "卒中后痴呆" OR "认知功能障碍" OR "混合性痴呆" OR "动脉硬化性痴呆" OR "丘脑性痴呆" OR "[关键部位](https://baike.baidu.com/item/%E5%85%B3%E9%94%AE%E9%83%A8%E4%BD%8D/8678986?fromModule=lemma_inlink)梗死性痴呆" OR "出血性痴呆" OR "多发性梗死痴呆" OR "多发性硬化性痴呆" OR "多发性梗塞痴呆" OR "宾斯旺格病" ) AND 主题:("临床" OR "疗效" OR "随机" OR "观察" OR "评价" OR "安全性" OR "有效性") |  |
| SinoMed | ("舒血宁注射液" [标题:智能] OR "舒血宁注射剂" [标题:智能] OR "银杏叶提取物" [标题:智能] OR "银杏叶注射液" [标题:智能] OR "银杏叶制剂" [标题:智能] OR "银杏叶注射剂" [标题:智能] OR "舒血宁" [标题:智能]) AND ("认知障碍" [标题:智能] OR "血管性痴呆" [标题:智能] OR "卒中后痴呆" [标题:智能] OR "认知功能障碍" [标题:智能] OR "混合性痴呆" [标题:智能] OR "动脉硬化性痴呆" [标题:智能] OR "丘脑性痴呆" [标题:智能] OR "[关键部位](https://baike.baidu.com/item/%E5%85%B3%E9%94%AE%E9%83%A8%E4%BD%8D/8678986?fromModule=lemma_inlink)梗死性痴呆" [标题:智能] OR "出血性痴呆" [标题:智能] OR "多发性梗死痴呆" [标题:智能] OR "多发性硬化性痴呆" [标题:智能] OR "多发性梗塞痴呆" [标题:智能] OR "宾斯旺格病" ) AND ("临床"[标题:智能] OR "疗效"[标题:智能] OR "随机"[标题:智能] OR "观察"[标题:智能] OR "评价"[常用字段:智能] OR "安全性"[常用字段:智能] OR "有效性"[常用字段:智能]) |  |
| Web of Science | TS=(Cognitive Dysfunctions OR Cognitive Impairments OR Cognitive Decline OR Vascular Dementia OR Binswanger Disease OR Vascular cognitive impairment OR Vascular cognitive disorders OR Vascular neurocognitive disorders OR Vascular cognitive disorders OR Vascular neurocognitive disorder OR Vascular mild cognitive impairment OR Post stroke cognitive impairment OR Post stroke dementia OR Subcortical vascular dementia OR Cerebrovascular cognitive impairment OR Subcortical ischemic vascular disease OR Multi-Infarct Dementias OR Dementia Multi-Infarct OR Dementia Multi Infarct) AND TS=(Shuxuening Injection OR Shuxuening OR *Ginkgo* OR *Ginkgo* *biloba* OR ginkgolide OR Yinxing OR Yinxingye) AND TS=(randomized controlled trial OR randomized clinical trial OR randomized trial OR clinical trial OR randomized controlled trial OR randomized clinical trial OR randomized trial OR clinical trial) |  |
| PubMed | #1 "Cognitive dysfunction"[Mesh Terms] OR "Cognitive Impairments"[Title/Abstract] OR "Vascular Cognitive Impairments"[Title/Abstract] OR "Mild Cognitive Impairment"[Title/Abstract] OR "Cognitive Decline"[Title/Abstract] OR "Vascular cognitive disorders" OR "Vascular neurocognitive disorder" OR "Vascular mild cognitive impairment" OR "Post stroke cognitive impairment"  #2 "Dementia, vascular"[Mesh Terms] OR "Vascular Dementia"[Title/Abstract] OR "Binswanger Disease"[Title/Abstract] OR "Post stroke dementia"[Title/Abstract]  #3 "Dementia, Multi-Infarct"[Mesh Terms] OR "Multi-Infarct Dementias"[Title/Abstract] OR "Dementia Multi-Infarct"[Title/Abstract] OR "; Dementia Multi Infarct"[Title/Abstract]  #4 "*Ginkgo*"[Title/Abstract] OR "*Ginkgo* *biloba*"[Title/Abstract] OR "ginkgolide"[Title/Abstract] OR "Yinxing"[Title/Abstract] OR "Yinxingye"[Title/Abstract] OR "Shuxuening"[Title/Abstract] OR "Shuxuening Injection"[Title/Abstract]  #5 "randomized controlled trial"[Publication type] OR randomized clinical trial[Publication type] OR randomized trial[Publication type] OR clinical trial[Publication type] OR "randomized controlled trial"[Title/Abstract] OR randomized clinical trial[Title/Abstract] OR randomized trial[Title/Abstract] OR clinical trial[Title/Abstract]  #6 (#1 OR #2 OR #3) AND #4AND #5 |  |
| Embase | ('multiinfarct dementia'/exp OR 'dementia, multi-infarct':ti,ab,kw OR 'dementia, multiinfarct':ti,ab,kw OR 'dementia, vascular':ti,ab,kw OR 'lacunar dementia':ti,ab,kw OR 'multi-infarct dementia':ti,ab,kw OR 'multi-infarction dementia':ti,ab,kw OR 'multiinfarction dementia':ti,ab,kw OR 'vascular dementia':ti,ab,kw OR 'multiinfarct dementia':ti,ab,kw OR 'cognitive defect'/exp OR 'cognition disorder':ti,ab,kw OR 'cognition disorders':ti,ab,kw OR 'cognitive complaints':ti,ab,kw OR 'cognitive decline':ti,ab,kw OR 'cognitive defects':ti,ab,kw OR 'cognitive deficiency':ti,ab,kw OR 'cognitive deficit':ti,ab,kw OR 'cognitive difficulties':ti,ab,kw OR 'cognitive disability':ti,ab,kw OR 'cognitive disorder':ti,ab,kw OR 'cognitive disorders':ti,ab,kw OR 'cognitive disturbance':ti,ab,kw OR 'cognitive dysfunction':ti,ab,kw OR 'cognitive impairment':ti,ab,kw OR 'cognitive problems':ti,ab,kw OR 'delirium, dementia, amnestic, cognitive disorders':ti,ab,kw OR 'overinclusion':ti,ab,kw OR 'response interference':ti,ab,kw OR 'cognitive defect':ti,ab,kw) AND ('*Ginkgo*'/exp OR '*Ginkgo* *biloba*':ti,ab,kw OR 'ginkgolide':ti,ab,kw OR 'Yinxing':ti,ab,kw OR 'Yinxingye':ti,ab,kw OR 'Shuxuening':ti,ab,kw OR 'Ginaton':ti,ab,kw) AND ('randomized controlled trial':ti,ab,kw OR 'randomized clinical trial':ti,ab,kw OR 'randomized trial':ti,ab,kw) |  |
| Cochrane Library | "Cognitive Dysfunctions" OR "Cognitive Impairments" OR "Cognitive Decline" OR "Vascular Dementia" OR "Binswanger Disease" OR "Vascular cognitive impairment" OR "Vascular cognitive disorders" OR "Vascular neurocognitive disorders" OR "Vascular cognitive disorders" OR "Vascular neurocognitive disorder" OR "Vascular mild cognitive impairment" OR "Post stroke cognitive impairment" OR "Post stroke dementia" OR "Subcortical vascular dementia" OR "Cerebrovascular cognitive impairment" OR "Subcortical ischemic vascular disease" OR "Multi-Infarct Dementias" OR "Dementia Multi-Infarct" OR "Dementia Multi Infarct" in Title Abstract Keyword AND "*Ginkgo*" OR "*Ginkgo* *biloba*" OR "ginkgolide" OR "Yinxing" OR "Yinxingye" OR "Shuxuening" OR "Shuxuening Injection" in Title Abstract Keyword - in Trials (Word variations have been searched) |  |
| **Abbreviation:** CNKI, China National Knowledge Infrastructure Database, VIP, VIP Database for Chinese Technical Periodicals, Wanfang, Wanfang Database, SinoMed, Chinese Biomedical Literature Database | | |

# Supplementary Table 4 Rules for assessment of the certainty of evidence

| Element | Criteria for downgrade |
| --- | --- |
| Study design | If the majority of the information based on the revised Cochrane Risk of Bias tool assessment was rated as moderate, the evidence was downgraded by one level. If the majority was rated as high, the evidence was downgraded by two levels |
| Inconsistency | If heterogeneity tests showed I2 exceeding 75%, the evidence was downgraded by two levels. If I2 was exceeding 50% and less than75%, the evidence was downgraded by one level |
| Indirectness | Assessment included several components: Population differences, intervention differences, outcome measurement differences, and indirect comparisons. If there was a serious suspicion regarding the directness of the evidence, the evidence was downgraded by one level. If there was a very serious suspicion regarding the directness of the evidence, the evidence was downgraded by two levels |
| Imprecision | If the 95% confidence interval crossed the null line, the evidence was downgraded by one level. For binary variables, when the total number of events was less than 300, and for continuous variables, when the total number of participants was less than 400, the evidence was downgraded by one level |
| Number of studies | If the number of studies was less than or equal to three, the evidence was downgraded by one level |

# Supplementary Table 5 Comparisons of the outcomes

| Outcomes | No. of studies | No. of participants | Statistical method | Effect size | I^2^ |
| --- | --- | --- | --- | --- | --- |
| MMSE | 20 | 2198 | MD (IV, Fixed, 95% CI) | 3.61 [3.06, 4.17] | 0 |
| HDS | 3 | 464 | MD (IV, Fixed, 95% CI) | 1.30 [0.21, 2.39] | 0 |
| BI | 6 | 626 | MD (IV, Fixed, 95% CI) | 9.06 [4.66, 13.45] | 0 |
| Overall response rate | 19 | 1899 | RR (M-H, Fixed, 95% CI) | 1.27 [1.21, 1.33] | 0 |
| NIHSS | 3 | 365 | MD (IV, Fixed, 95% CI) | -6.17[-7.90, -4.45] | 0 |
| Adverse Events | 4 | 384 | MD (IV, Fixed, 95% CI) | 0.72[0.36, 1.44] | 26 |

**Abbreviations**: MMSE, Mini-Mental State Examination; HDS, Hasegawa Dementia Scale; BI, Barthel Index; NIHSS, National Institutes of Health Stroke Scale; RR, risk ratio; M-H, Mantel-Haenszel; CI, confidence intervals; MD, mean difference; IV, inverse variance

# Supplementary Material 6 Sensitivity analyses for primary and secondary outcomes


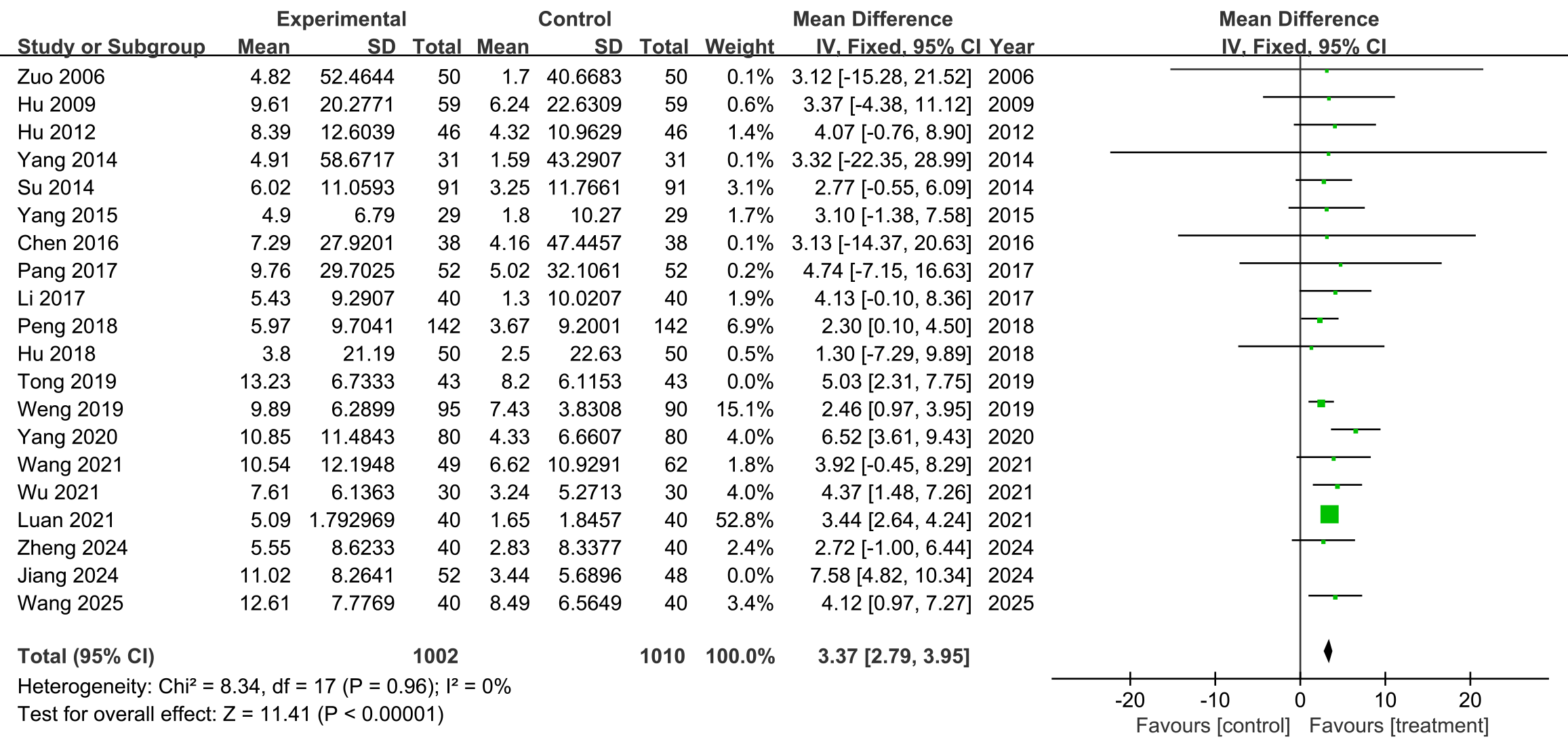


Supplementary Figure 1. Sensitivity analysis of MMSE.

Forest plot showing the sensitivity analysis for MMSE after exclusion of studies at high risk of bias.
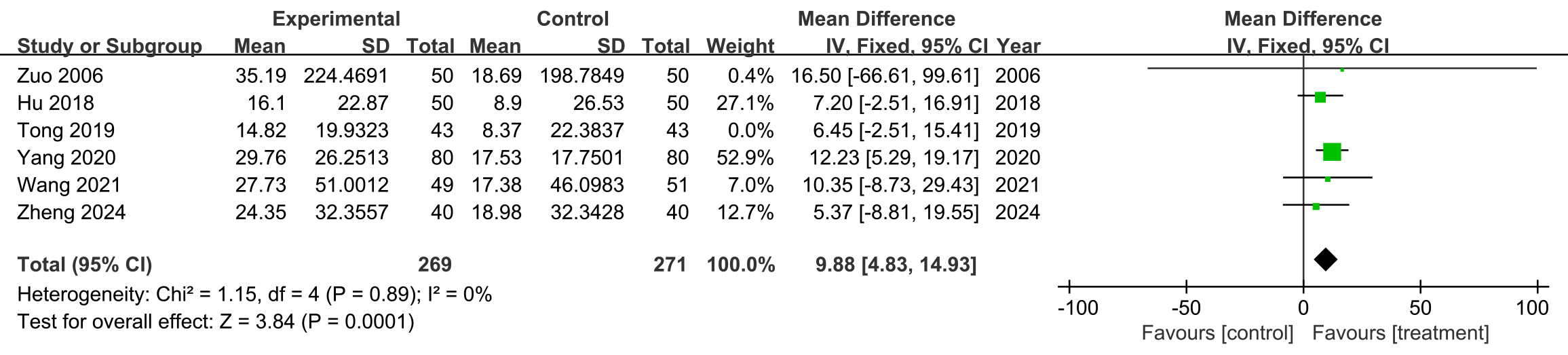


Supplementary Figure 2. Sensitivity analysis of BI.

Forest plot showing the sensitivity analysis for BI after exclusion of studies at high risk of bias.
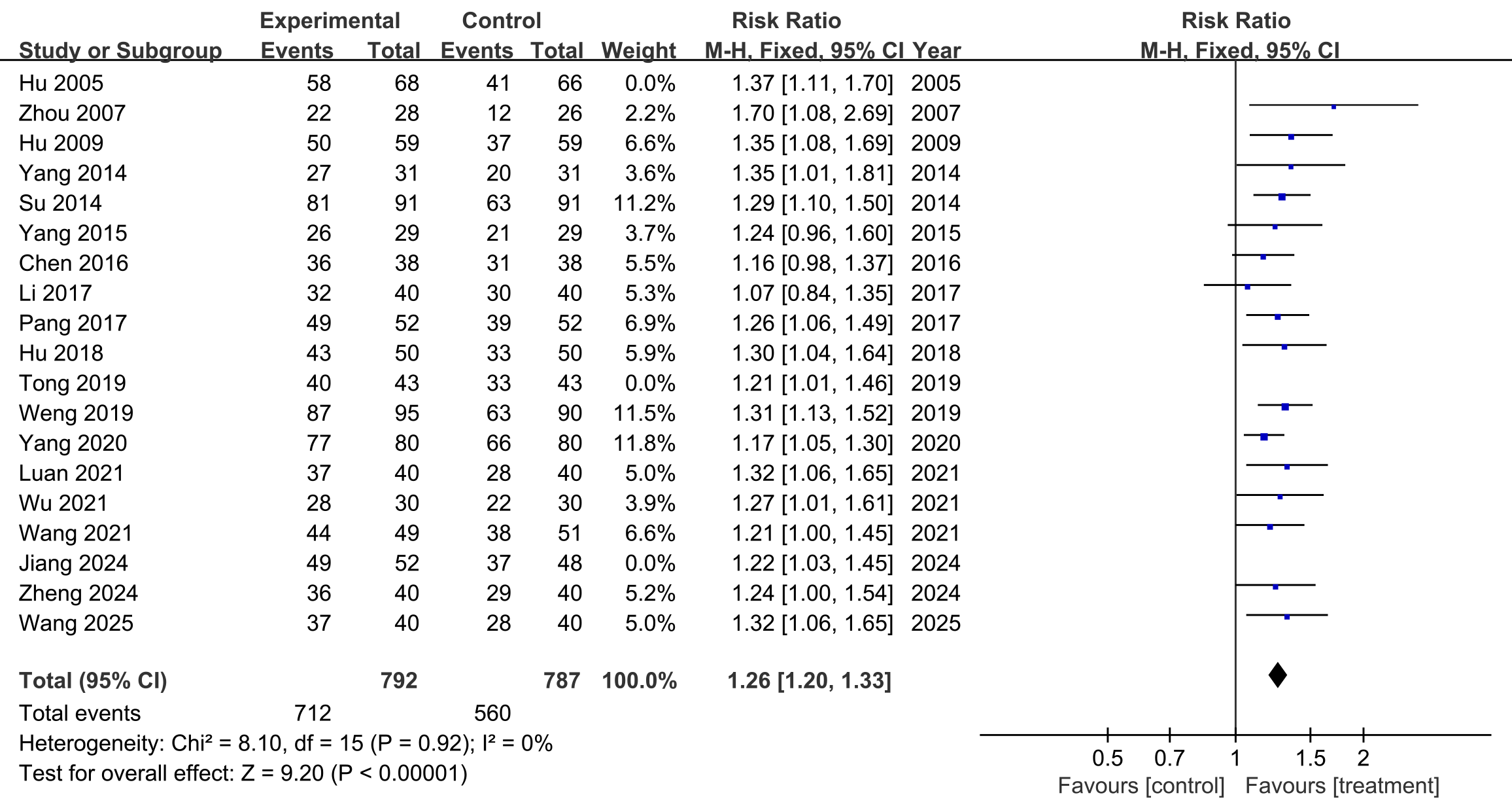


Supplementary Figure 3. Sensitivity analysis of overall response rate.

Forest plot showing the sensitivity analysis for overall response rate after exclusion of studies at high risk of bias.


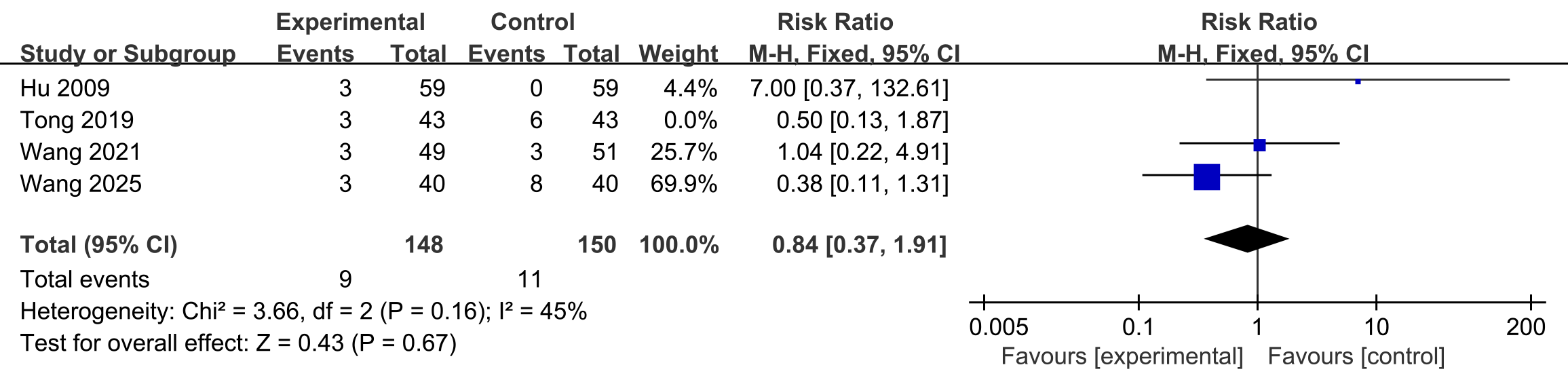


Supplementary Figure 4. Sensitivity analysis of adverse events.
Forest plot showing the sensitivity analysis for adverse events after exclusion of studies at high risk of bias.

# Supplementary Table 7 The information on **SXNI** from the included studies

| Study | Manufacturer | Botanical drug | Marker metabolites reported in study | Regulatory approval and labeled composition | Additional product information | Chemical analysis reported? (Y/N) |
| --- | --- | --- | --- | --- | --- | --- |
| (Hu, 2005) | Wanrong Sanjiu Pharmaceutical Co., Ltd. | *Ginkgo biloba* L., leaf extract | total flavonol glycosides; ginkgolides | NMPA Approval No. Z14021871; each 2 mL ampoule contains 1.68 mg total flavonol glycosides and 0.28 mg ginkgolides | Dilute ethanol extraction of *Ginkgo biloba* L. leaves followed by purification using macroporous adsorption resin and further refinement; excipients include ethanol and vitamin C. | N |
| (Zuo, 2006) | Beijing Shuanghe Pharmaceutical Co., Ltd. | *Ginkgo biloba* L., leaf extract | total flavonol glycosides; ginkgolides | NMPA Approval No. Z11021351; each 2 mL ampoule contains 1.68 mg total flavonol glycosides and 0.28 mg ginkgolides | Dilute ethanol extraction of *Ginkgo biloba* L. leaves followed by purification using macroporous adsorption resin and further refinement; excipients include ethanol and glucose. | N |
| (Zhou and Zhang, 2007) | NR | *Ginkgo biloba* L., leaf extract | total flavonol glycosides; ginkgolides | NR | NR | N |
| (Hu, 2009) | NR | *Ginkgo biloba* L., leaf extract | total flavonol glycosides; ginkgolides | NR | NR | N |
| (Hu et al., 2012) | NR | *Ginkgo biloba* L., leaf extract | total flavonol glycosides; ginkgolides | NR | NR | N |
| (Yang and Liang, 2014) | Shineway Pharmaceutical Group Co., Ltd. | *Ginkgo biloba* L., leaf extract | total flavonol glycosides; ginkgolides | NMPA Approval No. Z13020795; each 5 mL ampoule contains 4.2 mg total flavonol glycosides and 0.7 mg ginkgolides | Dilute ethanol extraction of *Ginkgo biloba* L. leaves followed by purification using macroporous adsorption resin and further refinement; excipients include sorbitol, 95% ethanol, and methionine. | N |
| (Su and Shang, 2014) | Tonghua Guhong Pharmaceutical Co., Ltd. | *Ginkgo biloba* L., leaf extract | total flavonol glycosides; ginkgolides | NMPA Approval No. Z22026295; each 5 mL ampoule contains 4.2 mg total flavonol glycosides and 0.7 mg ginkgolides | Dilute ethanol extraction of *Ginkgo biloba* L. leaves followed by purification using macroporous adsorption resin and further refinement; excipients include propylene glycol, ethanol, sorbitol, disodium edetate, and water for injection. | N |
| (Yang, 2015) | Beijing Shuanghe Pharmaceutical Co., Ltd. | *Ginkgo biloba* L., leaf extract | total flavonol glycosides; ginkgolides | NMPA Approval No. Z11021351; each 2 mL ampoule contains 1.68 mg total flavonol glycosides and 0.28 mg ginkgolides | Dilute ethanol extraction of *Ginkgo biloba* L. leaves followed by purification using macroporous adsorption resin and further refinement; excipients include ethanol and glucose. | N |
| (Chen, 2016) | Shineway Pharmaceutical Group Co., Ltd. | *Ginkgo biloba* L., leaf extract | total flavonol glycosides; ginkgolides | NMPA Approval No. Z13020795; each 5 mL ampoule contains 4.2 mg total flavonol glycosides and 0.7 mg ginkgolides | Dilute ethanol extraction of *Ginkgo biloba* L. leaves followed by purification using macroporous adsorption resin and further refinement; excipients include sorbitol, 95% ethanol, and methionine. | N |
| (Li et al., 2017) | NR | *Ginkgo biloba* L., leaf extract | total flavonol glycosides; ginkgolides | NR | NR | N |
| (Pang et al., 2017) | Heilongjiang Zhenbaodao Pharmaceutical Co., Ltd. | *Ginkgo biloba* L., leaf extract | total flavonol glycosides; ginkgolides | NMPA Approval No. Z23022003; each 2 mL ampoule contains 1.68 mg total flavonol glycosides and 0.28 mg ginkgolides | Dilute ethanol extraction of *Ginkgo biloba* L. leaves followed by purification using macroporous adsorption resin and further refinement; excipients include sorbitol and ethanol. | N |
| (Hu, 2018) | CSPC Yinhu Pharmaceutical Co., Ltd. | *Ginkgo biloba* L., leaf extract | total flavonol glycosides; ginkgolides | NMPA Approval No. Z14021945; each 5 mL ampoule contains 4.2 mg total flavonol glycosides and 0.7 mg ginkgolides | Dilute ethanol extraction of *Ginkgo biloba* L. leaves followed by purification using macroporous adsorption resin and further refinement; excipients include ethanol and vitamin C. | N |
| (Peng, 2018) | CSPC Yinhu Pharmaceutical Co., Ltd. | *Ginkgo biloba* L., leaf extract | total flavonol glycosides; ginkgolides | NMPA Approval No. Z14021945; each 5 mL ampoule contains 4.2 mg total flavonol glycosides and 0.7 mg ginkgolides | Dilute ethanol extraction of *Ginkgo biloba* L. leaves followed by purification using macroporous adsorption resin and further refinement; excipients include ethanol and vitamin C. | N |
| (Tong, 2019) | Shineway Pharmaceutical Group Co., Ltd. | *Ginkgo biloba* L., leaf extract | total flavonol glycosides; ginkgolides | NMPA Approval No. Z13020795; each 5 mL ampoule contains 4.2 mg total flavonol glycosides and 0.7 mg ginkgolides | Dilute ethanol extraction of *Ginkgo biloba* L. leaves followed by purification using macroporous adsorption resin and further refinement; excipients include sorbitol, 95% ethanol, and methionine. | N |
| (Weng, 2019) | Shineway Pharmaceutical Group Co., Ltd. | *Ginkgo biloba* L., leaf extract | total flavonol glycosides; ginkgolides | NMPA Approval No. Z13020795; each 5 mL ampoule contains 4.2 mg total flavonol glycosides and 0.7 mg ginkgolides | Dilute ethanol extraction of *Ginkgo biloba* L. leaves followed by purification using macroporous adsorption resin and further refinement; excipients include sorbitol, 95% ethanol, and methionine. | N |
| (Yang, 2020) | NR | *Ginkgo biloba* L., leaf extract | total flavonol glycosides; ginkgolides | NR | NR | N |
| (Wang, 2021) | NR | *Ginkgo biloba* L., leaf extract | total flavonol glycosides; ginkgolides | NR | NR | N |
| (Wu et al., 2021) | Chongqing Zein Pharmaceutical Co., Ltd. | *Ginkgo biloba* L., leaf extract | total flavonol glycosides; ginkgolides | NR | NR | N |
| (Luan, 2021) | NR | *Ginkgo biloba* L., leaf extract | total flavonol glycosides; ginkgolides | NR | NR | N |
| (Jiang, 2024) | NR | *Ginkgo biloba* L., leaf extract | total flavonol glycosides; ginkgolides | NR | NR | N |
| (Zheng et al., 2024) | NR | *Ginkgo biloba* L., leaf extract | total flavonol glycosides; ginkgolides | NR | NR | N |
| (Wang and Li, 2025) | Heilongjiang Zhenbaodao Pharmaceutical Co., Ltd. | *Ginkgo biloba* L., leaf extract | total flavonol glycosides; ginkgolides | NMPA Approval No. Z23022003; each 2 mL ampoule contains 1.68 mg total flavonol glycosides and 0.28 mg ginkgolides | Dilute ethanol extraction of *Ginkgo biloba* L. leaves followed by purification using macroporous adsorption resin and further refinement; excipients include sorbitol and ethanol. | N |

**Abbreviations**: NR, not reported; NMPA,National Medical Products Administration
