# Supplementary figures and images for: Efficacy and safety of Ginkgo biloba leaf extract injection for vascular cognitive impairment: a systematic review and meta-analysis
Source: Front Pharmacol. 2026 Apr 13;17:1720444. doi: 10.3389/fphar.2026.1720444 (PMC13111442; doi:10.3389/fphar.2026.1720444)

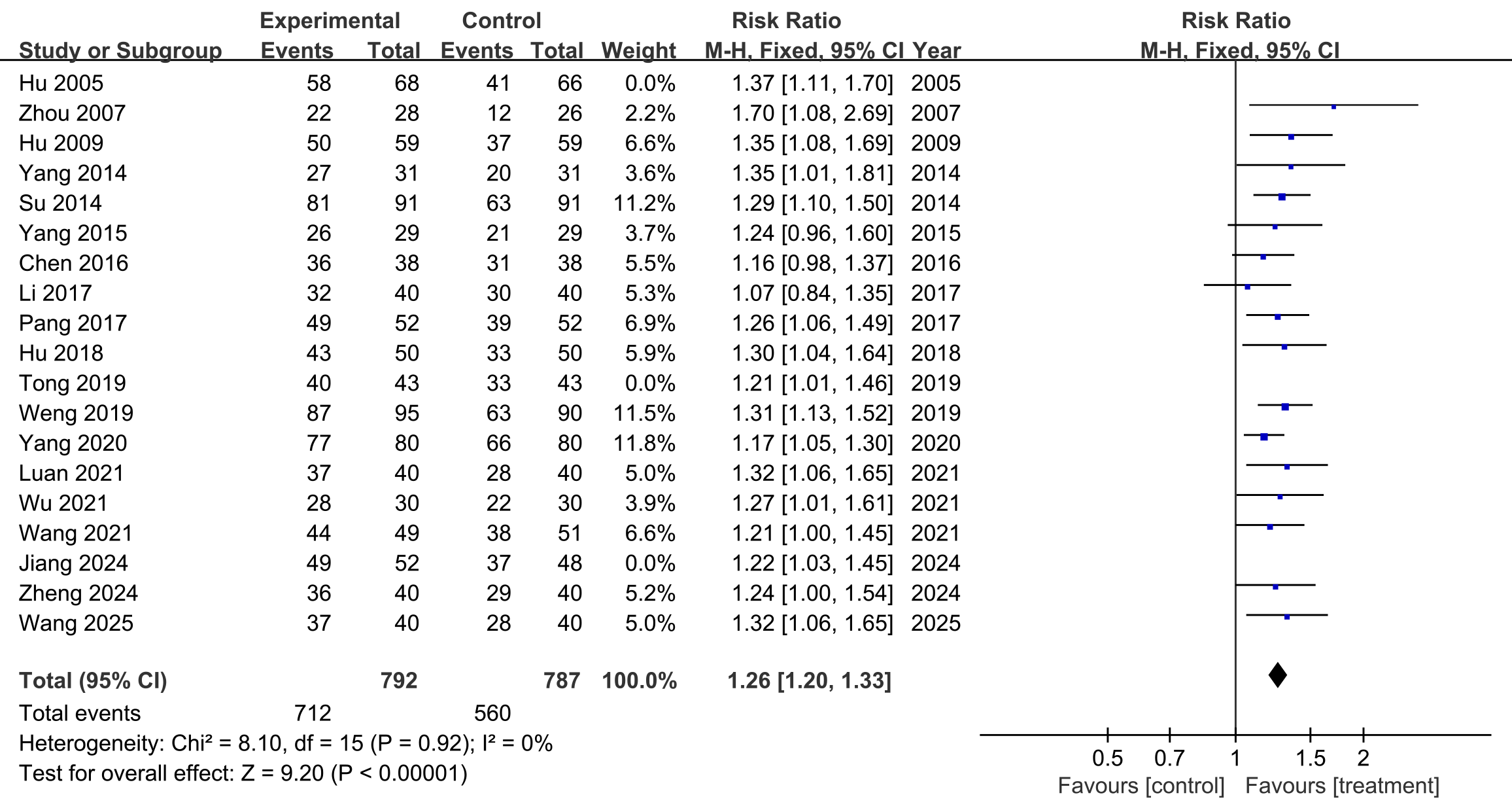

Supplement: Supplementary file 2 [file Image3.tif]

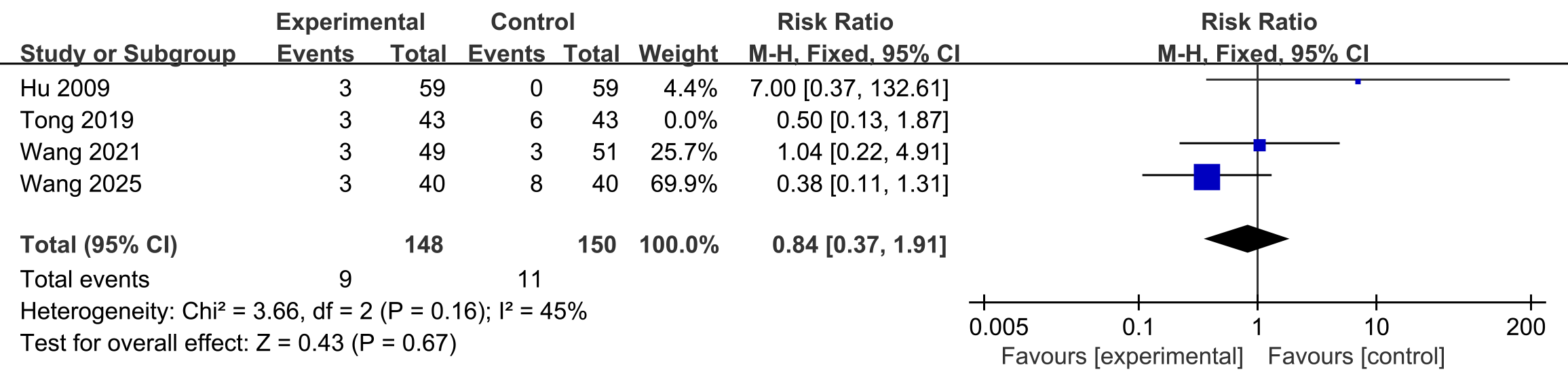

Supplement: Supplementary file 3 [file Image4.tif]

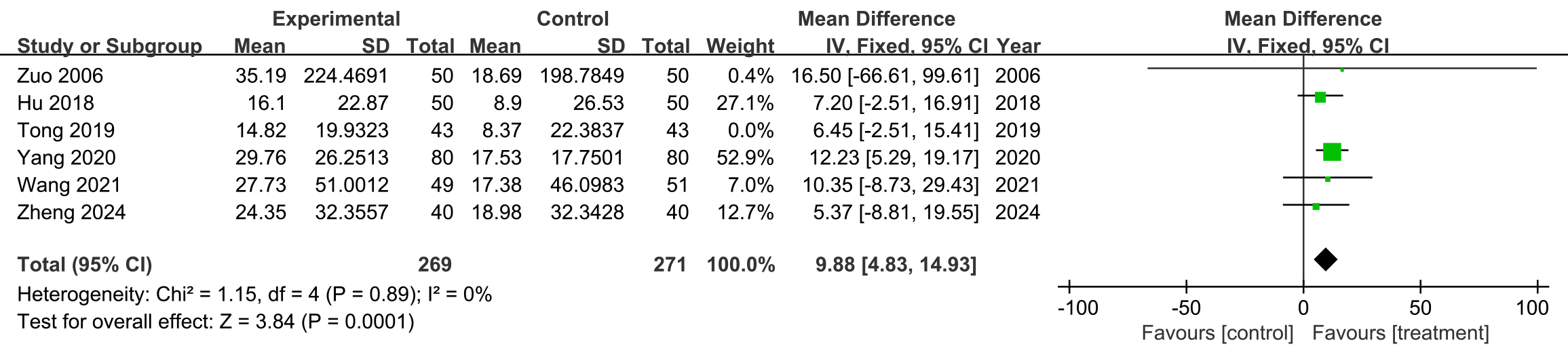

Supplement: Supplementary file 4 [file Image2.tif]

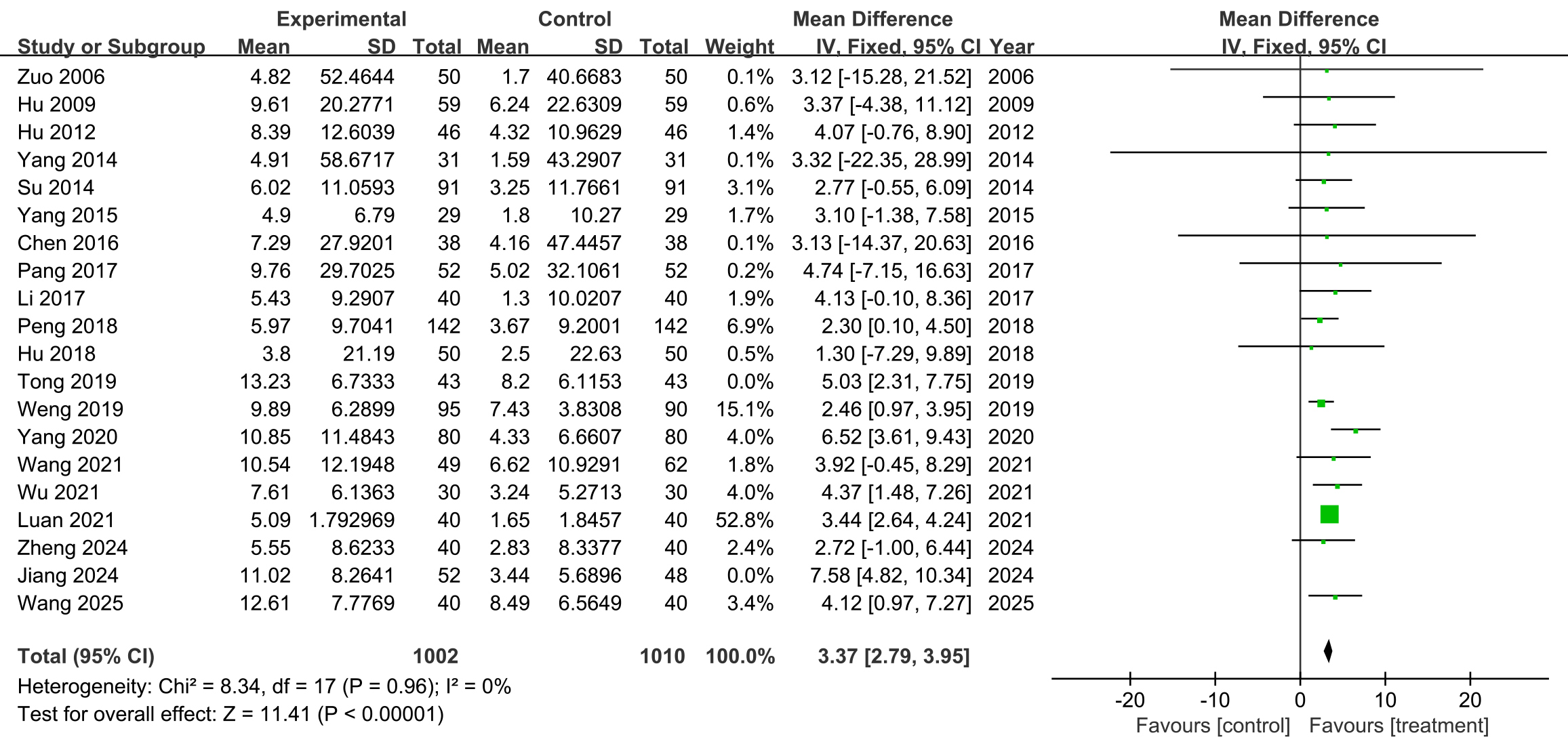

Supplement: Supplementary file 5 [file Image1.tif]
